# Helical tomotherapy: an innovative radiotherapy technique for the treatment of locally advanced oropharynx and inoperable oral cavity carcinoma

**DOI:** 10.1186/1748-717X-8-210

**Published:** 2013-09-10

**Authors:** Vittorio Donato, Michele Cianciulli, Sofia Fouraki, Leonardo Vigna, Alberto Rocco, Nicola Raffetto, Gianluca Bellocchi

**Affiliations:** 1 Department of Radiation Oncology, San Camillo-Forlanini Hospital, Rome, Italy; 2 Department of Radiation Oncology, University “Sapienza”, Rome, Italy; 3 Department of Medical Oncology, San Camillo-Forlanini Hospital, Rome, Italy; 4 Department of Otorhinolaryngology, San Camillo-Forlanini Hospital, Rome, Italy

**Keywords:** Helical tomotherapy, Oropharyngeal cancer, Oral cavity cancer, Intensity-modulated radiotherapy, Concurrent chemoradiation

## Abstract

**Background:**

To report our initial clinical experience of helical tomotherapy (HT) in the treatment of locally advanced oropharynx and inoperable oral cavity cancer.

**Methods:**

Between February 2008 and January 2011, 24 consecutive patients, 15 with oropharyngeal cancer and 9 with oral cavity cancer were treated with exclusive radiotherapy or concomitant chemoradiotherapy. Simultaneous integrated boost (SIB) in 30 fractions scheme was prescribed to all patients, using Helical Tomotherapy. Doses administered to primary tumor, oropharynx/oral cavity and positive lymph-nodes and negative lymph-nodes were 66–67.5 Gy, 60–63 Gy and 54 Gy, respectively.

**Results:**

Complete response rate for the oropharynx and the oral cavity group was 86.7% and 77.8%, respectively. The 1 and 2-year Overall Survival (OS) and Disease Free Survival (DFS) rate for the oropharynx group was 92.9%, 85.1%, 92.9% and 77.4% respectively. For the oral cavity group, 1 and 2-year OS and DFS rates were 55.6%, 55.6%, 75% and 75%, respectively. No patient developed grade ≥3 mucositis, dysphagia or dermatitis. The maximum late-toxicity grade observed was 2, for all the variables examined.

**Conclusions:**

HT appears to achieve encouraging clinical outcomes in terms of response, survival and toxicity rates.

## Introduction

Carcinomas of the oropharynx and oral cavity are two of the most common types of head and neck squamous cell carcinoma (HNSCC), which is the sixth leading cancer by incidence worldwide [1]. HNSCCs are strongly associated with certain lifestyle risk factors such as tobacco smoking and alcohol consumption [2,3]. Oropharyngeal infection by oncogenic type-16 human papillomavirus (HPV) has been also associated with oropharyngeal squamous cell carcinoma [4].

Although survival rates for HNSCC are improving, locoregional control remains suboptimal, especially in patients with advanced-stage disease; in this setting, treatment should involve a multidisciplinary team approach (head and neck surgeon, medical oncologist, radiation oncologist). Regarding oral cavity carcinoma, surgery has been and still remains the mainstay of treatment for resectable tumors, reserving radiation therapy for the advanced stages. On the other hand, chemoradiotherapy or radiotherapy can be used as exclusive treatment, either for initial or advanced-stage oropharyngeal cancer, in order to preserve functional anatomy.

During the last decade, radiation oncology has witnessed an explosion in innovation of treatment modalities: intensity modulated radiotherapy (IMRT) allows to deliver a high radiation dose to the tumor, with improved target conformality and surrounding healthy tissue sparing, in comparison with three-dimensional (3D) plans [5-7].

Helical tomotherapy (HT) is an innovative radiotherapy technique, which integrates linear accelerator and computerized tomography (CT) technology to deliver IMRT in an helical pattern, thanks to a continuously rotating gantry. The integrated image-guidance system provides daily 3D imaging of the tumor, achieving precise irradiation, and therefore decreased toxicity to healthy tissue, with possible treatment adaption (Image Guided Radiotherapy or IGRT) [8,9].

A tomotherapy Hi-Art® system was introduced into clinical routine at the Department of Radiation Oncology, San Camillo-Forlanini Hospital, Rome, Italy, in January 2008. This article reports our initial clinical experience of HT, in the treatment of patients with locally advanced oropharynx and inoperable oral cavity carcinoma, in terms of response, acute and late toxicity rates.

## Materials and methods

### Patient and tumor characteristics

Between February 2008 and September 2011, a total of 105 head and neck cancer patients (60 men and 55 women, median age 62 years old, range 33–86) were treated with HT, either definitively or postoperatively, at San Camillo-Forlanini Hospital of Rome. The present study group consists of 24 consecutive patients, 15 with oropharyngeal cancer (tonsil, *n* = 9, base of tongue, *n* = 4, soft palate, *n* = 2) and 9 with oral cavity cancer (retromolar trigone, *n* = 4, floor of mouth, *n* = 3, oral tongue, *n* = 2), treated with exclusive radiotherapy or concomitant chemoradiotherapy, from February 2008 to January 2011 (Table 1). There were 15 males and 9 females with a median age of 58 years old (range 45–85) and an *Eastern Cooperative Oncology Group Performance Status* (ECOG PS) as follows: ECOG 1, *n* = 4 patients; ECOG 2, *n* = 18 patients; ECOG ≥ 3, *n* = 2 patients. The staging evaluation included: clinical examination, fiberoptic endoscopic evaluation, Positron Emission Tomography (PET)/ Magnetic Resonance Imaging (MRI) scans of the head and neck region, chest X-ray, complete blood counts, liver and renal function tests and dental evaluation.

**Table 1:** Patient characteristics

**Total patients**	**24**	**(100%)**
**Gender**	*n*	%
Male	15	(62.5%)
Female	9	(37.5%)
**Age**	*years*	
Median age	58	
Range	45–85	
**ECOG PS**	*n*	%
0	0	(0%)
1	4	(16.7%)
2	18	(75%)
≥3	2	(8.3%)
**Subsite**	*n*	%
Tonsil	9	(37.5%)
Base of tongue	4	(16.7%)
Soft palate	2	(8.3%)
Retromolar trigone	4	(16.7%)
Floor of mouth	3	(12.5%)
Oral tongue	2	(8.3%)
**Pathology**	*n*	%
Squamous cell carcinoma	24	(100%)

*Abbreviations:ECOG PS* Eastern Cooperative Oncology Group Performance Status.

Concerning clinical stage, 20 patients (83.3%) had a IVA stage disease and 4 patients (16.7%) a III stage, according to the 7th Edition of the American Joint Committee on Cancer (AJCC). Twenty one patients had an advanced primary tumor stage (T3, *n* = 41.6%, T4a, *n* = 45.8%) and were node positive (Table 2). All patients provided written informed consent.

**Table 2:** Tumor stage

**Disease stage**	***n***	**%**
Stage III	4	(16.7%)
Stage IVA	20	(83.3%)
**Primary tumor stage**	*n*	%
T1	1	(4.2%)
T2	2	(8.3%)
T3	10	(41.7%)
T4a	11	(45.8%)
T4b	0	(0%)
**Regional lymph node stage**	*n*	%
N0	3	(12.5%)
N1	3	(12.5%)
N2a	2	(8.3%)
N2b	2	(8.3%)
N2c	14	(58.3%)
N3	0	(0%)

### Radiotherapy treatment planning

Patients were immobilized in the supine position, with the neck hyper-extended, using a head rest and custom thermoplastic mold, achieving head, neck and shoulders immobilization. During the simulation process, CT images indexed every 5-mm were obtained with a CT scanner for treatment planning. Target volumes and normal structures were contoured on a Pinnacle® treatment planning system and MRI/PET images were fused with the CT images, in order to delineate the gross tumor volume (GTV). This latter was defined as the volume containing the visible on imaging and/or clinically detectable tumor, while the GTV-node, as any lymph nodes over 10 mm in short axis dimension or smaller nodes with necrotic centres or rounded contours. Planning target volume (PTV) was obtained adding 3 mm margins to clinical target volume (CTV). Brainstem was contoured as an organ at risk (OAR) with a maximum dose of 54 Gy. Spinal cord was outlined with a 3 mm isocentric margin, also (maximum dose 45 Gy). Other critical organs included: optic chiasm and optic nerve (maximum dose 45 Gy), mandible (maximum dose 70 Gy), inner ear (mean dose <50 Gy) and both parotid glands. These latter are responsible for 60% to 65% of the saliva produced and xerostomia is a major acute and late side effect that can have a significant negative impact on a patient’s quality of life; in order to limit this kind of toxicity, particular attention was paid during treatment planning, achieving a mean dose <26 Gy.

Radiotherapy was delivered once daily, five days a week, using a tomotherapy Hi-Art® machine. Megavoltage CT acquisitions were performed before treatment in all patients for setup verification. Simultaneous integrated boost (SIB) in 30 fractions scheme was prescribed to all patients.

Patients treated with exclusive radiotherapy received a dose of 67.5 Gy in 2.25 Gy daily fractions for tumor and 63 Gy in 2.1 Gy daily fractions for oropharynx/oral cavity and positive lymph nodes. Lower doses were used for patients treated with concomitant chemotherapy: 66 Gy in 2.2 Gy daily fractions for tumor and 60 Gy in 2 Gy daily fractions for oropharynx/oral cavity and positive lymph nodes. Negative lymph nodes were irradiated with equal dose for both groups: 54 Gy in 1.8 Gy daily fractions.

### Chemotherapy

Of the 24 patients, 15 (oropharyngeal carcinoma, *n* = 10, oral cavity carcinoma, *n* = 5) received concomitant platinum-based chemotherapy. Patients over 80 years of age or those with a poor ECOG performance status and/or affected by comorbid conditions, received only radiotherapy. Subsite of tumor distribution of chemotherapy-treated patients was as follows: tonsil, *n* = 7, base of tongue, *n* = 3, retromolar trigone, *n* = 2, floor of mouth, *n* = 2, oral tongue, *n* = 1. Chemotherapy (either carboplatin 100mg/m [2] or cisplatin 80 mg/m [2]) was given intravenously every 3 weeks in an outpatient setting. Taking into account the fact that we used an hypofractionated regime, a lower dosage of chemotherapy (cisplatin 80 mg/mq instead of 100 mg/q) was administrated in order to contain toxicity.

### Clinical assessment

Patients were assessed at least weekly during radiotherapy by a radiation oncologist and every 2 weeks by an otolaryngologist for adverse effects. Toxicity was scored according to the Common Terminology Criteria for Adverse Effects, version 4.0. Acute-toxicity assessment, performed during the radiotherapy treatment, included mucositis, dysphagia and dermatitis. Late-toxicity assessment, performed during the follow-up visits, included xerostomia, pain, trismus, fibrosis and neck edema.

Response at treatment was evaluated 3 months after the completion of radiotherapy, on the basis of a clinical examination and CT or MRI scans. Complete response was defined as the disappearance of tumor and lymph node involvement, while partial response as the decrease in the multiplication product of the two largest diameters of tumor and/or positive lymph nodes by at least 50%.

After completion of treatment, follow-up visit, performed once a month the first 6 months, every 3 months the first 2 years and every 6 months thereafter, included: physical examination, MRI/PET scans and fiberoptic endoscopic examination. The median follow-up period was 24 months (range 3–53).

### Statistical methods

The statistical method used was the Kaplan- Meier survival analysis. For the calculation of Disease Free Survival, the event was defined as a) reccurrence of disease or b) cancer-related death. Death not related to cancer was not considered as event.

## Results

Twenty-three out of 24 patients (95.8%) received the complete course of planned radiotherapy. Only one patient did not complete the radiation therapy course because of clinical deterioration, requiring recovery. No patient required the insertion of nasogastric or percutaneous endoscopic gastrostomy (PEG) tube for nutritional support during radiotherapy.

### Treatment outcome

**Figure 1:**
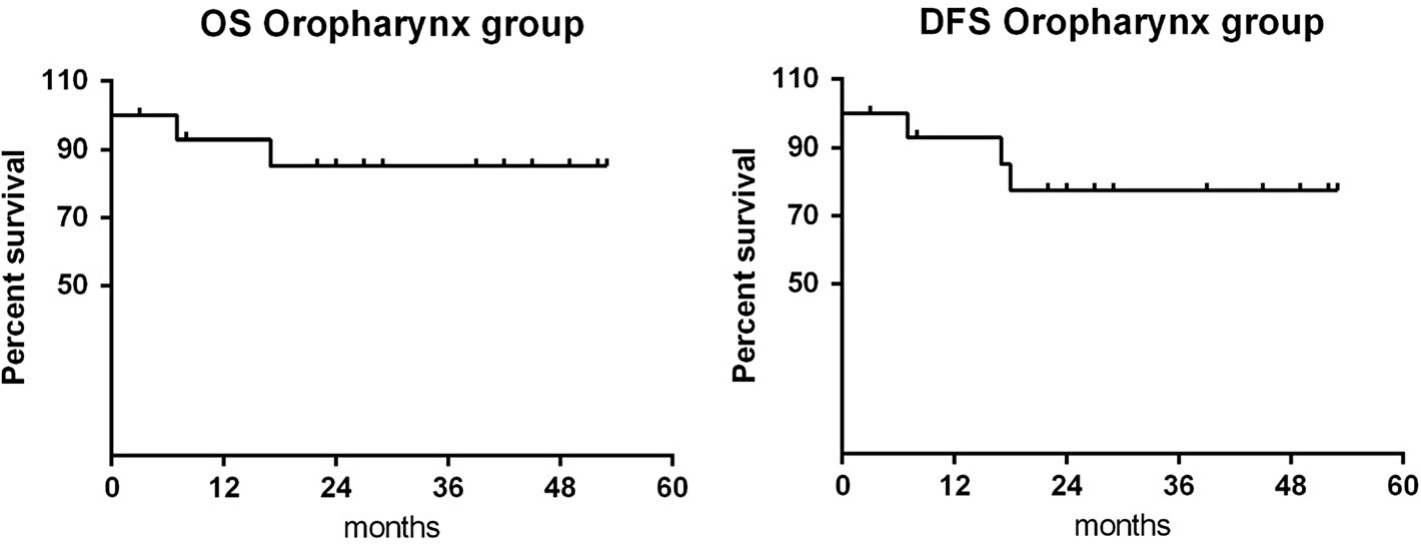
Overall survival and disease-free survival of the oropharynx group.

From the analysis of oropharynx cancer patients, a complete response was observed in 13 out of 15 patients (86.7%). The 1 and 2-year overall survival (OS) rate was 92.9% and 85.1%, respectively, while the 1 and 2-year disease-free survival (DFS) rate was 92.9% and 77.4%, respectively (Figure 1). One patient died of disease progression, 2 of concurrent disease (myocardial infarction, pulmonary embolism), while a fourth patient, who had a partial response, died 17 months after the completion of radiotherapy due to heart failure. There was no evidence of distant metastases. Relapse of disease has been observed only in one patient (subsite: base of tongue, stage IVA), 40 months after the end of radiotherapy.

**Figure 2:**
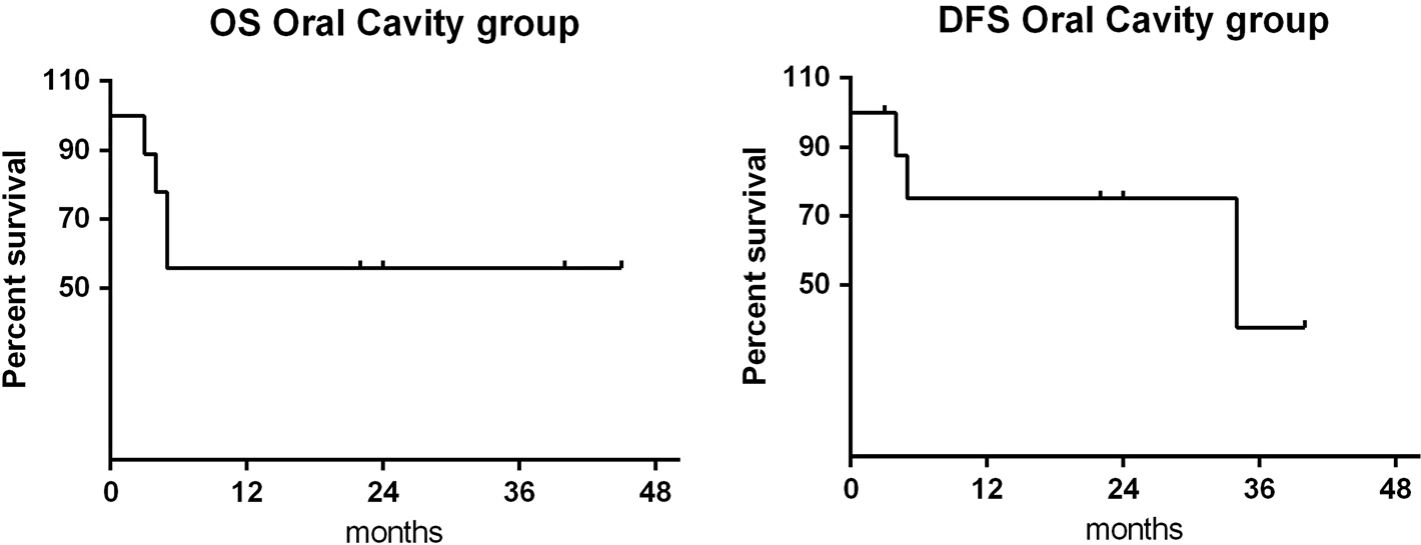
Overall survival and disease-free survival of the oral cavity group.

From the analysis of oral cancer patients, a complete response was observed in 7 out of 9 patients (77.8%). The 1 and 2-year OS rate was 55.6%, while the 1 and 2-year DFS rate was 75% (Figure 2). Only one patient presented distant metastases (lymph node and pulmonary), 34 months after the completion of radiotherapy. Two patients died of disease progression and 2 of concurrent disease (liver disease, intestinal perforation).

### Acute and late toxicity

Concerning acute-toxicity, grade 1 and grade 2 mucositis was observed in 12.5% and 87.5 % of patients, respectively. As for dysphagia, 70.8% of patients developed grade 2 toxicity, while the rest of them (29.2%) grade 1. Regarding dermatitis, grade 1 toxicity was observed in 54.2% of patients and grade 2 in the rest of them (45.8%). No patient developed grade ≥3 mucositis, dysphagia or dermatitis (Table 3). Late-toxicity was evaluated in 18 patients, as in 2 patients were clinical assessment was not feasible and 4 died shortly (within 6 months) after the completion of treatment. The maximum grade late-toxicity observed was grade 2 for all the adverse effects examined; many patients, did not develop toxicity at all, for certain variables (Table 4). Median time of evaluation of xerostomia was 24 months. Mild xerostomia (grade 1) was the most frequent late-toxicity effect (11 patients, 61.1%). Six patients did not experience xerostomia at all, while 1 patient complained grade 2 xerostomia. Regarding pain symptom, 61.1% of patients did not experience pain, while 4 of them (22.2%) experienced grade 1 and, 3 patients (16.7%) grade 2 pain. Trismus was absent in 77.8% of patients; only 4 patients (22.2%) developed trismus, grade 1. Late fibrosis (grade 1) was present in 10 patients (56.5%) and absent in the rest of them. Only 7 patients (38.9%) developed neck edema (grade 1).

**Table 3:** Acute toxicity rates

**Mucositis**	***n***	**%**
Grade 1	3	(12.5%)
Grade 2	21	(87.5%)
Grade 3	0	(0%)
Grade 4	0	(0%)
Grade 5	0	(0%)
**Dysphagia**	*n*	%
Grade 1	7	(29.2%)
Grade 2	17	(70.8%)
Grade 3	0	(0%)
Grade 4	0	(0%)
Grade 5	0	(0%)
**Dermatitis**	*n*	%
Grade 1	13	(54.2%)
Grade 2	11	(45.8%)
Grade 3	0	(0%)
Grade 4	0	(0%)
Grade 5	0	(0%)

**Table 4:** Late toxicity rates

**Xerostomia**	***n***	**%**
Absent	6	(33.3%)
Grade 1	11	(61.1%)
Grade 2	1	(5.6%)
Grade 3	0	(0%)
Grade 4	0	(0%)
Grade 5	0	(0%)
**Fibrosis**	*n*	%
Absent	10	(55.5%)
Grade 1	8	(44.5%)
Grade 2	0	(0%)
Grade 3	0	(0%)
Grade 4	0	(0%)
Grade 5	0	(0%)
**Trismus**	*n*	%
Absent	14	(77.8%)
Grade 1	4	(22.2%)
Grade 2	0	(0%)
Grade 3	0	(0%)
**Pain**	*n*	%
Absent	11	(61.1%)
Grade 1	4	(22.2%)
Grade 2	3	(16.7%)
Grade 3	0	(0%)
**Neck edema**	*n*	%
Absent	11	(61.1%)
Grade 1	7	(38.9%)
Grade 2	0	(0%)
Grade 3	0	(0%)

## Discussion

In the treatment of oropharyngeal/oral cavity carcinoma, locoregional control is one of the most important goals to achieve, as local recurrences are common and represent a frequent cause of death. Considering the natural history of disease and the relatively low incidence of distant metastases, an effective local control could be translated in a higher possibility of cure. However, obtainment of a satisfactory local control requires high radiation doses to the target volume and, consequently, appropriate sparing of surrounding normal tissue, in order to minimize acute and late toxicity.

HT is an advanced IGRT technique that permits accurate delivery of a high, conformal dose to the target volume from a rotational gantry. Its additional option in inverse planning optimization, results in a more uniform dose to the tumor and a better avoidance of organs at risk, thus, in a higher locoregional control probability with decreased toxicity rates.

The present study, regarding our initial experience of HT in the treatment of patients with locally advanced oropharynx and inoperable oral cavity carcinoma, provided encouraging results in terms of response, survival and toxicity rates.

The evidence of literature concerning IMRT for orophanyngeal carcinoma, reports 2-year locoregional tumor control rates of 90 to 98% for patient populations consisting mainly of stage III–IV disease [10]. However, these series contain both definitively and postoperatively treated patients, and variable ratios of patients treated with concurrent chemoradiotherapy or radiotherapy alone. Chao et al. [11] compared IMRT to conventional radiotherapy techniques, in the treatment of oropharyngeal carcinoma with definitive radiotherapy; they reported a 2-year locoregional control and DFS rate of 68% and 58% for the conventional radiotherapy treated group, and 88% and 80% rate for the IMRT treated group, respectively.

The M D Anderson Cancer Center (MDACC) experience in the treatment of oropharyngeal carcinoma with definitive IMRT, reported a 4-year estimate of DFS, locoregional control and distant metastasis-free survival of 66%, 78% and 84%, respectively [12].

Shueng et al [13] published the preliminary results of their experience of concurrent chemoradiotherapy for oropharyngeal cancer using HT. Ten patients were treated with concomitant chemoradiotherapy to doses of 70 Gy, 63 Gy and 56 Gy to the GTV, high-risk subclinical area and low-risk subclinical area, respectively. The actuarial OS, DFS, locoregional control and distant metastasis-free survival rates at 18 months were 67%, 70%, 80% and 100%, respectively. No grade 3 toxicity for dermatitis and body weight loss and only one instance of grade 3 mucositis were noted.

Despite the clear efficacy of a combined-modality approach in locally advanced oral/oropharyngeal squamous cell carcinoma, toxicity can be considerable [14]. Long-term xerostomia is one of the most inconvenient side effects. Sheng et al. [15] and Van Vulpen et al. [16] analysed the advantages of sparing parotid glands, concluding that HT could simultaneously reduce parotid normal tissue complication probability and maintain similar target dose homogeneity. The results obtained in the present study confirmed this benefit regarding patient salivary function.

The *Memorial Sloan-Kettering Cancer Center* experience [17], in the treatment of oropharyngeal cancer patients with IMRT, reported grade 3 dermatitis and mucositis rates of 6% and 38%, respectively. Bhide et al [18] compared the response of oral and pharyngeal mucosa in patients receiving concomitant chemoradiotherapy with IMRT technique for head and neck cancer, using hypofractionated accelerated schemes of 2.17 Gy, 2.25 Gy and 2.4 Gy per fraction. Grade 3 dysphagia was correlated with the length of pharyngeal mucosa receiving doses close to the prescription dose; its incidence was lower and patients recovered earlier in case of greater overall treatment time.

In our current study complete response, 2-year OS and 2-year DFS rates in patients affected by locally advanced oropharyngeal carcinoma were 86.7%, 85.1% and 77.4%, respectively. In terms of clinical characteristics, it’s necessary to underline that many patients presented several negative features. In particular, there were 2 patients over age 80 years, 20 (83.3%) with an ECOG PS ≥2 and many suffering from important comorbid conditions. This relates with the relative low number of patients not candidable to chemotherapy. In this report, HPV status was not analyzed as it is not yet used to guide treatment, except in the context of a clinical trial. When available is valuable prognostically, being a favorable factor if positive. It could be hypothesized that part of the favorable outcome in the oropharynx group can be attributed to HPV positivity. Concerning clinical stage, 20 patients (83.3%) were classified as stage IVA and 21 (87.5%) had positive lymph nodes (with bilateral involvement in 14, 58.3%). Compliance to the treatment was excellent: all patients except one, completed planned radiotherapy, while no patient presented the necessity to feed by tube or PEG. Regarding acute effects, no grade ≥3 toxicity was registered; grade 2 mucositis, dysphagia and dermatitis rates were 87.5%, 70.8% and 45.8%, respectively. The overall absence of higher grade acute mucositis could be due to daily MVCT of Helical Tomotherapy for set up verification, pharmacological therapy administered for symptoms prevention and frequent clinical checks. As for late toxicity, only one patient experienced grade 2 xerostomia.

The optimal management of oral cavity squamous cell carcinoma typically requires surgical resection followed by adjuvant radiotherapy or chemoradiotherapy, in the setting of adverse pathologic features. Published IMRT outcomes specific to this disease subsite are poor, although post-operative IMRT is frequently used to treat oral cavity cancers.

Hsieh CH et al. [19], who treated high-risk oral cavity carcinoma patients with HT in a postoperative setting, reported 2-year overall survival, disease-free survival, locoregional control and distant metastasis-free rates of 94%, 84%, 92% and 94%, respectively; grade 3 mucositis, dermatitis and leucopenia rates were 42%, 5% and 5%, respectively.

In contrast, outcomes of locally advanced oral cavity carcinoma patients treated with definitive radiotherapy, seem to be less successful, compared to adjuvant radiotherapy treatment. Sher et al [20], reported a 2-year actuarial overall survival and local control rate of 85% and 91%, for adjuvant IMRT and 63% and 64%, for definitive IMRT, respectively.

On the basis of this evidence, we adopted an hypofractionated regimen, with a higher radiobiological equivalent total dose to the primary tumor, in order to improve local control and survival rates in patients treated with exclusive radiotherapy. In our study, complete response, 2-year OS and 2-year DFS rates in inoperable oral cavity carcinoma, were 77.8%, 55.6% and 75%, respectively.

## Conclusions

Concomitant radiochemotherapy or exclusive radiotherapy represents an important therapeutic option and a valid alternative to surgery, in patients affected by locally advanced oropharynx and inoperable oral cavity carcinoma. Helical tomotherapy allows a high radiation dose delivery to the target volume and therefore, an increased probability of local control and improved survival rates. Moreover, it’s capability to create highly conformal dose distributions permits significant sparing of surrounding organs at risk, decreasing the probability of acute and late toxicity effects. The present study, provided encouraging results in terms of response, survival and toxicity rates, however, a long-term follow-up and a larger number of patients are necessary, in order to confirm these preliminary findings.

## Competing interests

The authors declare that they have no competing interests.

## Authors’ contribution

VD supervised the study. MC was the main investigator, designed the study, and collected the data. SF helped the main investigator to analyze the data and wrote the first draft. LV, AR, NR and GB contributed to the analysis, critically evaluated the paper, and provided the final draft. All authors read and approved the final revision of the manuscript.
